# Optical logic array: a photonic solution towards universal computing

**DOI:** 10.1007/s12200-024-00145-z

**Published:** 2024-12-25

**Authors:** Lu Fang

**Affiliations:** Department of Electronic Engineering, Tsinghua University, Beijing, 100084 China

## Abstract

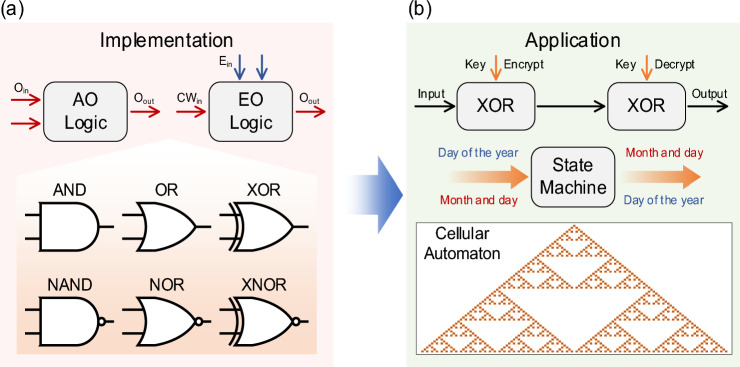

Logic operations are the cornerstone of modern computation, underpinning almost every aspect of information processing. As the development of electronic arithmetic logical unit approaches physical limits, it is crucial to explore new solutions to sustain the rapid growth in computing resources requirements. Optical logic, leveraging the unique properties of light in terms of speed and efficiency, offers significant advantages over traditional electronic counterparts. Light inherently supports ultrafast signal propagation and enables high-bandwidth communication. Its capability to process information in parallel through wavelength or spatial division multiplexing further enhances its computational potential. These attributes not only make optical logic a potential solution to overcome the capacity limitations of electronic circuits, but also unlock a wealth of opportunities in optical information processing.

Optical logic strives to implement logic gates in the optical domain and construct advanced logic circuits based upon them. State-of-the-art optical logic can be typically classified into all-optical (AO) and electro-optical (EO) schemes, as shown in Fig. 1a. The AO logic leverages optical nonlinear effects, or diffraction of light within optical media to perform logic operations. Well-known optical nonlinearities, like four-wave mixing and cross-phase modulation, have been widely employed in optical logic. Meanwhile, novel nonlinear effects have also been utilized to further enhance its performance. For example, Hui Li et al. presented a full set of temporal logic gates with ultrafast switching speed in localized exciton polaritons [1]. The implementation of logic with spatial light often relies on diffraction effects. Ryosuke Mashiko et al. implemented the 16 two-input logic operations by incorporating shadow casting and diffractive neural networks [2]. 256 sets of logic operands were executed simultaneously by leveraging the spatial parallelism of light. However, due to relatively low nonlinear efficiency and complex spatial encoding, AO schemes based on nonlinear effects or diffraction are difficult to achieve the large bit-width. On the other hand, EO logic performs logic operations with specially designed optical structures by loading electrical signals onto the optical carrier, thereby circumventing the need for the all-optical nonlinearity. Various logic functions, such as XOR/XNOR gates, 2–4 decoder and parity checker are realized by the EO method. In light of the superior performance of optical modulators, EO logic excels in both speed and energy efficiency. Zhoufeng Ying et al. pointed out the power consumption in electronic circuits has a cubic correlation to clock frequency while that in optical circuits has a linear correlation [3]. They demonstrated a 4-bit arithmetic EO logic unit with an operating frequency of 20 GHz and showed excellent energy efficiency compared to its electronic counterparts.

**Fig. 1:**
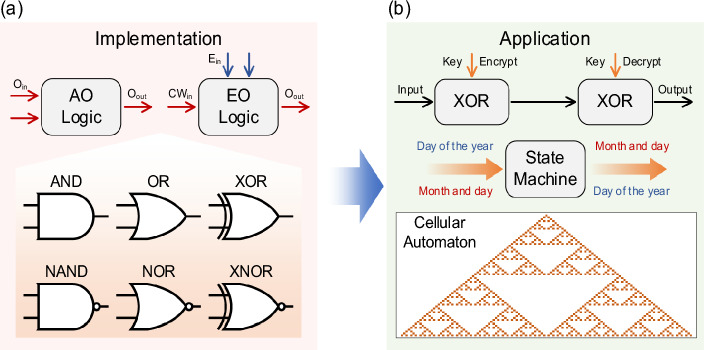
Schematic of optical logic computing: from **a** implementation to **b** application

The recent advances of optical logic arrays lead to representative applications in information encryption and decryption, image processing, etc. As shown in Fig. 1b, the input signal undergoes an XOR operation with the key to generate the encrypted information. To restore the original input signal, the exact same key must be used during the decryption process. Ting He et al*.* proposed on-chip optoelectronic logic gates and demonstrated imaging and image processing performed by these logic gates [4]. They utilized AND gate for symbol recognition, XOR gate for edge extraction, and OR gate for image fusion. To solve more complex problems, the scalability and high-throughput of optical logic arrays are urgently demanded.

Recently, the group of Xinliang Zhang and Jianji Dong from Huazhong University of Science and Technology have made a breakthrough by demonstrating a nine-input optical programmable logic array based on parallel spectrum modulation [5]. They established a linear relationship between the number of modulators and the input operands, utilizing both the wavelength and spatial dimensions to scale up the array, thereby enabling large-scale optical logic arrays. The substantial expansion of the optical logic array opens up exciting new possibilities for advanced applications. They successfully implemented state machines capable of deducing the date (month and day) from the input day of the year and performing the reverse process. Furthermore, they solved cellular automata with optical logic for the first time, running various evolution models. The proposed system achieved lower complexity in simulation and configuration, coupled with high computing speeds (~ 10 GHz). Notably, the Conway’s Game of Life was demonstrated, which, as a Turing-complete system, can simulate any algorithmically computable process given appropriate initial conditions. This progress highlights the growing potential of optical logic, indicating its gradual evolution towards general-purpose computing as the bit width continues to expand.

With the implementation of various optical logic circuits and the application of them in practical scenarios, optical logic rises as a promising photonic solution for universal computing. It is anticipated that the development of scalable, high-throughput and high-speed optical logic architectures would play an important role in shaping the future of information processing and computing technologies, revolutionizing next-generation computing systems.
